# Supplementary material for: Towards Routine Implementation of Liquid Biopsies in Cancer Management: It Is Always Too Early, until Suddenly It Is Too Late
Source: Diagnostics (Basel). 2021 Jan 11;11(1):103. doi: 10.3390/diagnostics11010103 (PMC7826562; doi:10.3390/diagnostics11010103)
Supplement: Supplementary file 1 [file diagnostics-11-00103-s001.pdf]

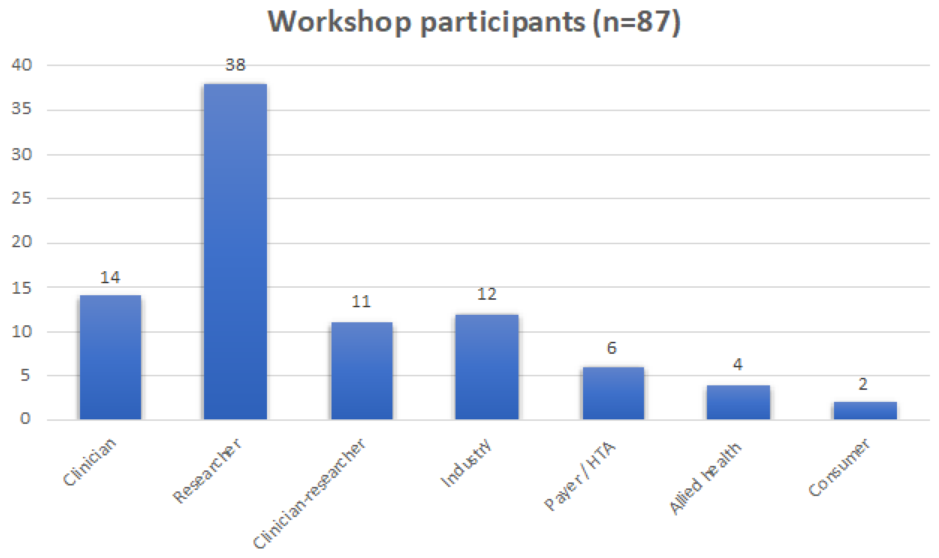

Figure S1. Workshop participants consenting to complete the pre-workshop survey.

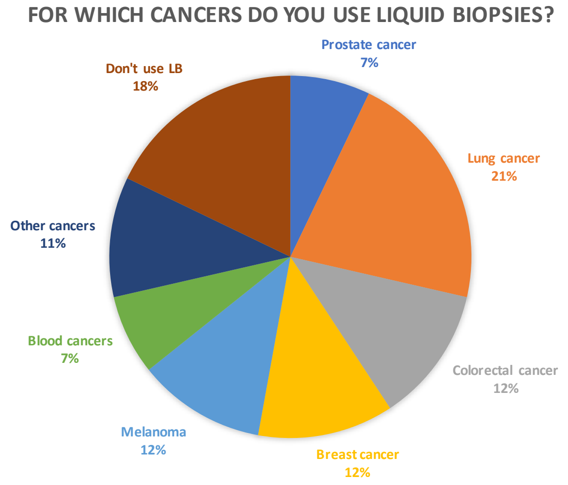

Figure S2. Overview of cancers for which liquid biopsies are used by workshop participants.

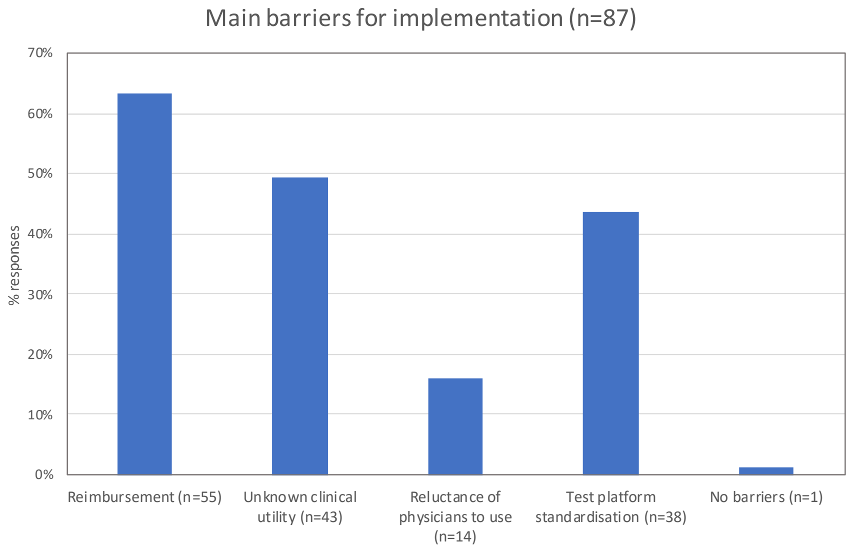

Figure S3. What are the main barriers for implementation of liquid biopsies (data from the pre-workshop survey).

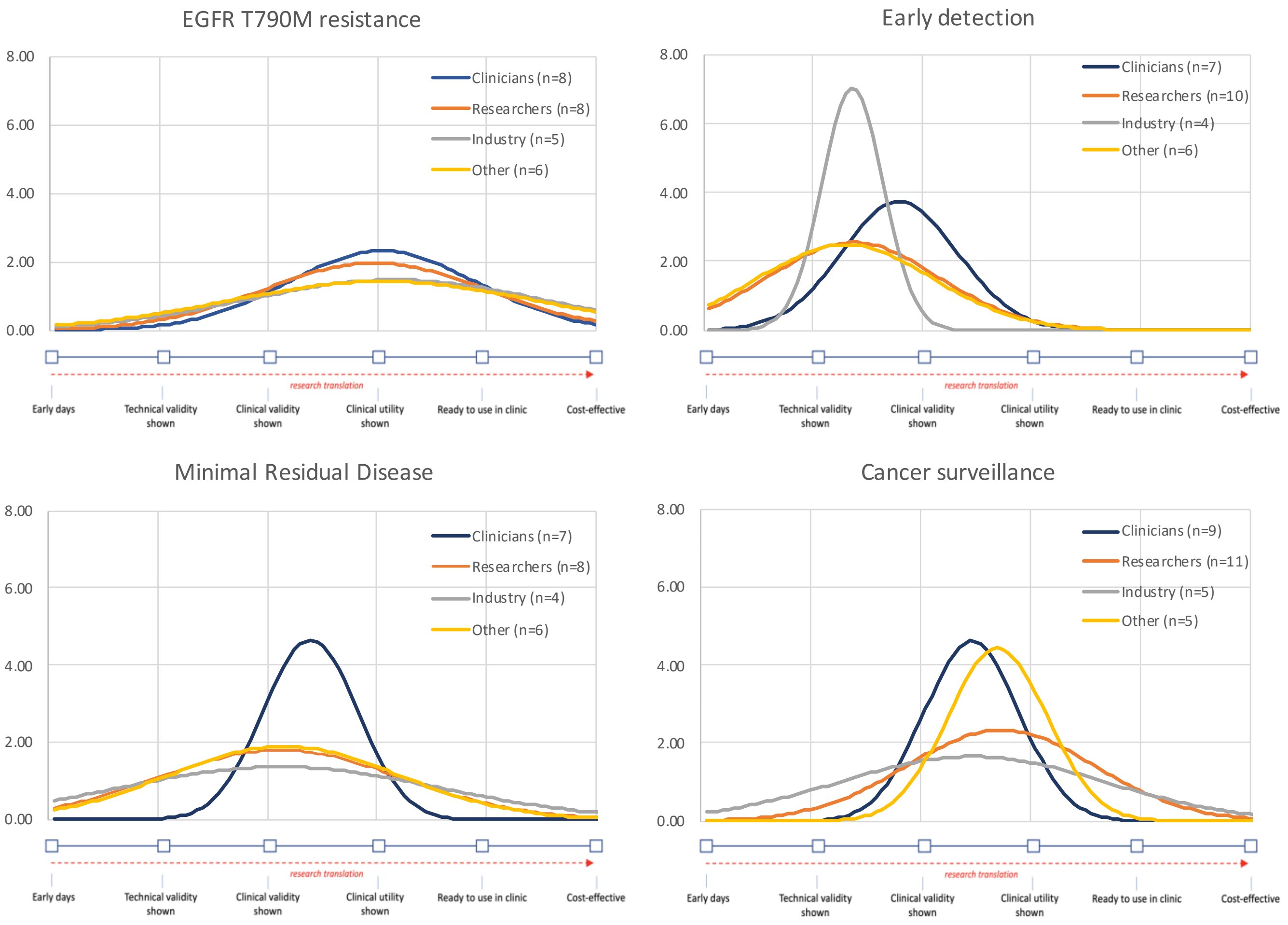

Figure S4. Probability of level of evidence available (%) for four different applications and according to four different stakeholder groups. The width of the distribution reflects the uncertainty of the respondents.
